# Nutrient scoring for the DEGS1-FFQ – from food intake to nutrient intake

**DOI:** 10.1186/s40795-022-00636-2

**Published:** 2023-01-13

**Authors:** Ronja Thieleking, Lennard Schneidewind, Arsene Kanyamibwa, Hendrik Hartmann, Annette Horstmann, A. Veronica Witte, Evelyn Medawar

**Affiliations:** 1 Max Planck Institute for Human Cognitive and Brain Sciences, Leipzig, Germany; 2 Department of Psychology and Logopedics, Faculty of Medicine, University of Helsinki, Helsinki, Finland; 3 Clinic for Cognitive Neurology, University Medical Center Leipzig, Leipzig, Germany

**Keywords:** Dietary assessment, Nutrient scoring, Fiber intake, FFQ, BMI

## Abstract

**Background:**

While necessary for studying dietary decision-making or public health, estimates of nutrient supply based on self-reported food intake are barely accessible or fully lacking and remain a challenge in human research. In particular, detailed information on dietary fiber is limited. In this study we introduce an automated openly available approach to assess self-reported nutrient intake for research purposes for a popular, validated German food frequency questionnaire (FFQ).

**Methods:**

To this end, we i) developed and shared a code for assessing nutrients (carbohydrates, fat, protein, sugar, fiber, etc.) for 53 items of the quantitative, validated German DEGS1-FFQ questionnaire implementing expert-guided nutritional values of diverse sources with several raters. In a sample of individuals (n_GUT-BRAIN_ = 61 (21 female) overweight, omnivorous), we ii) cross-validated nutrient intake of the last 7 days and the last 24 h and iii) computed test–retest reliability across two timepoints. Further, iv) we reported newly computed nutrient intake for two independent cross-sectional cohorts with continuous weight status and different dietary habits (n_Mensa_ = 134 (79 female, 1 diverse), n_GREADT_ = 76 male). Exploratively, we v) correlated computed, energy-adjusted nutrient intake with anthropometric markers and HbA1c and vi) used linear mixed models to analyse the predictability of BMI and WHR by nutrient intake.

**Results:**

In overweight adults (*n* = 61 (21 female), mean age 28.2 ± 6.5 years, BMI 27.4 ± 1.6 kg/m^2^) nutrient intakes were mostly within recommended reference nutrient ranges for both last 7 days and last 24 h. Recommended fiber intake was not reached and sugar intake was surpassed. Calculated energy intake was significantly higher from last 24 h than from last 7 days but energy-adjusted nutrient intakes did not differ between those timeframes. Reliability of nutrient values between last 7 days and 24 h per visit was moderate (Pearson’s rho_all_ ≥ 0.33, rho_max_ = 0.62) and absolute agreement across two timepoints was low to high for 7 days (Pearson’s rho_min_ = 0.12, rho_max_ = 0.64,) and low to moderate for 24 h (Pearson’s rho_min_ = 0.11, rho_max_ = 0.45). Associations of dietary components to anthropometric markers showed distinct sex differences, with overall higher intake by males compared to females and only females presenting a negative association of BMI with fiber intake. Lastly, in the overweight sample (but not when extending the analysis to a wider BMI range of 18.6–36.4 kg/m^2^), we could confirm that higher BMI was predicted by lower energy-adjusted fiber intake and higher energy-adjusted fat intake (when adjusting for age, sex and physical activity) while higher WHR was predicted by higher energy intake.

**Conclusion:**

We provide an openly available tool to systematically assess nutrient intake, including fiber, based on self-report by a common German FFQ. The computed nutrient scores resembled overall plausible and reliable measures of nutrient intake given the known limitations of FFQs regarding over- or underreporting and suggest valid comparability when adjusting for energy intake. Our open code nutrient scoring can help to examine dietary intake in experimental studies, including dietary fiber, and can be readily adapted to other FFQs. Further validation of computed nutrients with biomarkers and nutrient-specific metabolites in serum, urine or feces will help to interpret self-reported dietary intake.

**Supplementary Information:**

The online version contains supplementary material available at 10.1186/s40795-022-00636-2.

## Introduction

### Benefits and drawbacks of dietary habit assessment

Nutrition science relies on tracking dietary intake of individuals using more or less sophisticated self-reported dietary diaries, energy chambers or other observational measures [1]. Advantages of commonly used food frequency questionnaires (FFQ) based on self-report are low costs, low time investment and the possibility of self-administration. Disadvantages are non-uniformity across studies (due to differences in number and variety of food items, time frame of food intake), self-report of non-expert study participants leading to under-/over-/misreporting of food intake and lack of detail on specific food items [2]. In particular, in real-life settings self-reported measures are criticized for being too imprecise to carry valuable evidence, especially for providing robust data for nutritional epidemiological research and dietary recommendations for society [3], however methodological improvements such as ecological momentary assessment might overcome some of these limitations [4]. Self-administered questionnaires save valuable interviewing resources, but usually require more preparation time and pre-testing than an interview-administered FFQ. Participants may only report commonly eaten items or miss some of the questions, therefore checking for completeness and plausibility is necessary [5]. Computer-based methods for the recall of dietary intake offers the possibility to check the answers automatically and to implement a variety of quality controls to assist the overall standardization and accuracy of the collected data [6, 7]. In addition, those tools are more cost-effective, yet accuracy is hard to compare to conventional methods [7].

### Nutrient scoring for FFQs with a focus on fiber

Depending on the research question, dietary patterns or certain aspects of the diet, i.e. single macro- or micronutrients, may be important to assess reliably. Yet, extracting macro- and micronutrient levels from dietary data requires either using nutrient reference databases and developing a scoring method for the FFQ at hand or feeding the data manually into commercial software, resulting in high effort and error-prone methodology. Data on the nutrient level is crucial for assessing associations or effects of nutrient intake on health or behaviour. For example, dietary fiber is known to be a beneficial dietary component related to better health status [8], lower all-cause mortality [9], colorectal cancer [10], inflammatory bowel disease [11], and depression [12]. A systematic review of 185 prospective studies and 58 clinical trials with 4,635 adult participants suggested highest risk reduction with a daily fiber intake of 25 g to 29 g, in a dose-dependent response when combining dietary fiber and whole-grain foods [8]. Plant-based (vegetarian, vegan) diets are estimated to be on average higher in dietary fiber compared to animal-based diets [13, 14]. Strict plant-based diets have been shown to reach those beneficial fiber intake ranges [15], whereas gradual increases in fiber were shown depending on dietary adherence (meat-eaters, fish-eaters, vegetarians, vegans) [16]. Moreover, diets high in fat and sugar are in parallel likely to be low in fiber [17]. Indeed, measuring actual dietary fiber intake is difficult due to the definition of substances that fall under this category and how these can be accurately measured [18]. Dietary fiber can be defined as “non-digestible carbohydrates and lignin that are intrinsic and intact in plants” [10], whereas other sources define dietary fiber as non-digestible plant polysaccharides [19]. There are also different approaches to the categorization of dietary fiber, since it can be classified by its source [10, 20] or by its subtypes [20]. Most commonly, for fiber-specific FFQs, such as the EAT5 FFQ [21], dietary fiber intake FFQ (DFI-FFQ) [19] or others [22, 23], fiber is measured in gram per day [15, 19], or fiber intake relative to total energy intake, i.e. gram per 1000 kcal per day [10]. When analyzing a larger data set, it is reasonable to split the sample in quintiles based on fiber intake [10, 15], whereas smaller datasets are commonly split in tertiles or at the median. In addition, fiber intake was shown to be gender-specific [19]. More recent questionnaire development efforts also include the distinction of soluble, insoluble and prebiotic dietary fibers (FiberTAG, [24]). Overall, fiber-specific FFQs and fiber scoring could serve as a cost-efficient and quick tool to detect insufficient intake. Yet, they remain underdeveloped and niche due to low accuracy and low validity.

### Recall bias in FFQ data

Furthermore, FFQs are subject to systematic errors due to recall bias caused by misreporting of food intake. The degree of subjective bias is linked to the characteristics of the respondent. Reliable predictors for potential underreporting of food intake are age, BMI, and level of education. Levels of underreporting were 31% in the Second National Health and Nutrition Examination Survey and 46% for women and 29% for men in the National Diet and Nutrition Survey of British Adults [5]. Also, individuals with higher BMI underreported to a higher degree than those with lower BMI [25].

### Selecting and designing FFQs for the aim of the study

The most appropriate way to validate FFQs seems to combine dietary diary records and biomarkers [7]. Since biochemical measurements of nutrients provide unbiased estimates of dietary intake, they are not subject to recall bias. However, they are limited to certain nutrients and have inherent error sources which are linked to the biochemical assays themselves and the individual characteristics and metabolism of the participants. Most biomarkers do not allow assessment of true absolute dietary intake [5].

Despite being a validated and widely used tool for assessing dietary intake (e.g. [26–29]), the semi-quantitative DEGS1-FFQ is missing an automated nutrient scoring methodology for making nutrient intake assessment in Germany more feasible.

## Aim

The commonly used German DEGS1-FFQ, which was first used within the DEGS1 study [30, 31] by the Robert Koch Institute (Berlin, Germany), is a tool to measure the approximate intake of 53 single food items based on self-report of frequency and quantity. The main outcome is mean daily portion in grams for each of the 53 items. However, this measure is of limited use due to its numerous outcome variables and not suitable for investigating more specific intake of macro- and micronutrients and dietary fiber. The aim is to translate self-reported dietary intake (using the German DEGS1-FFQ) into nutrient intake per day for various nutrients of interest. We further assessed test–retest reliability for computed nutrient intake and assayed potential relations to anthropometric measures and biomarkers.

## Methods

### Study sample

The herein analyzed data has been taken from a within-subject cross-over design in a human dietary intervention study (title: GUT-BRAIN, registered under NCT03829189). Data for the validation of the nutrient scoring includes two baseline assessments second baseline after wash-out period). The main sample consists of n_GUT-BRAIN_ = 61 (21F) omnivorous participants with a mean age of 28.2 ± 6.5 years and an average BMI of 27.4 ± 1.6 kg/m^2^ (range: 25–31 kg/m^2^). Anthropometrics, namely BMI, Waist-to-hip ratio (WHR) and body fat mass, as well as blood pressure were measured at each visit. Participants gave their written informed consent before taking part in the study and were compensated with 9€ per hour.

### DEGS1-food frequency questionnaire

Food intake in the GUT-BRAIN study was recorded using the validated German DEGS1-FFQ [30] for the following time periods: a) the last 7 days (FFQ7d) and b) the last 24 h (FFQ24h) (n_FFQ7d_ = n_FFQ24h_ = 61 individuals, 110 datapoints). In general, the DEGS1-FFQ assesses the frequency and amount of 53 food items and groups consumed over a certain period. This period can vary from 4 weeks down to 24 h. The original scoring of the DEGS1-FFQ results in the mean daily portion of each food item in grams.

Participants in the GUT-BRAIN study completed both questionnaires at two visits respectively. Visits were at least 28 days apart. Each participant filled out the questionnaire online via browser-based LimeSurvey v3.0. The questionnaire as well as the.lsq-file for LimeSurvey can be accessed via https://gitlab.gwdg.de/omega-lab/ffq-nutrient-scoring or https://osf.io/h73wj/.

### Additional data samples

For cross-validation, we extended the data by two independent samples. The first sample, titled “Mensa”, consisted of n_Mensa_ = 134 (79 female, 43 male, 1 diverse, 11 NA) German university cafeteria visitors with a mean BMI of 22.5 ± 3.1 kg/m^2^ (range: 17.5–40.6 kg/m^2^). The study was an observational online study investigating post-meal ratings of hunger and well-being. Participants in the “Mensa” study were omnivorous dieters only. The DEGS1-FFQ in the “Mensa” study covered a time frame of 14 days dietary intake [32]. Secondly, in a cross-sectional sample of adult men including omnivorous and vegetarian dieters with a mean age of 26.6 ± 4.4 years (range: 18–40) and a mean BMI of 23.6 ± 2.7 kg/m^2^ (range: 18.6–36.4 kg/m^2^), dietary intake was assessed with the DEGS1-FFQ for the last 4 weeks (n_GREADT_ = 76 M) [33]. The “GREADT” study was designed with two groups with significantly different dietary fat and sugar intake as measured by the 26-item German Version of the Dietary Fat and Free Sugar-Short Questionnaire (DFS) [34]. HbA1c was assessed as a long-term marker of glucose metabolism.

### Nutrient database

All reference nutrient values were extracted from the German Nutrient Reference Database “Bundeslebensmittelschlüssel” (BLS, version 3.02, Max Rubner-Institut, Karlsruhe, Germany) and, in rare cases, from individual sources directly from food suppliers. The BLS is a database comprising extensive tables on food composition (macro- and micronutrients) of single food items.

### DEGS1-FFQ dietary scoring

As mentioned above, the original scoring of the DEGS1-FFQ [30] provides the mean daily portion for each of the 53 food items/groups in grams. The calculation of the mean daily portions is based on the amount and frequency that were indicated for each food item.

However, for 16 of the 53 food items/groups participants give more specific information which the original scoring disregards. These “type” questions provide information on the way a food item was processed (cooked or fried), if a food item was high or low in fat or if a certain drink was consumed undiluted or diluted. Our aim was to incorporate these details to obtain more precise information on the participants food intake. Therefore, we evaluated the answers of the “type” questions in order to correctly calculate the nutrients of interest of the daily food intake.

In order to convert the daily food intake into nutrient values, we created a reference table. This reference table includes nutrient values for all 53 food items/groups as well as for the variations of the food items addressed by the “type” questions. The macronutrient values cover energy (kcal/100 g), protein, fat, carbohydrates, dietary fiber and overall sugar as well as the fiber subclasses cellulose, lignin, water-soluble and -insoluble fiber. Other nutrients include tyrosine, tryptophan, saturated fatty acids, short-, medium- and long-chain fatty acids as well as Omega-3 and Omega-6 fatty acids. All nutrient values except for energy are provided in mg/100 g.

We published the creation process of the reference table, the LimeSurvey questionnaire files and the R-code containing the nutrient scoring here: https://gitlab.gwdg.de/omega-lab/ffq-nutrient-scoring.

The steps from the raw DEGS1-FFQ data to macro- and micronutrients consumed per day, are the following:

- calculation of the mean daily portions for each of the 53 food items/groups based on the original scoring
- identifying additional information specified in the 16 “type” questions
- calculation of the nutrients of interest for each food item/group by combining all acquired information on the 53 food items
- summing up all nutrients of interest to acquire respective total intake per day
- remove outliers based on energy values (if desired)
- energy-adjustment of nutrient values according to the residual method [35]

### Statistical analysis

All analyses were performed with R (version 3.6.1). Figures were created with the packages ggplot2 (version 3.3.0) and corrplot (version 0.9.0).

### Main analysis

Outliers regarding energy values were removed according to interquartile range rule (above Q3 + 1.5xIQR or below Q1—1.5xIQR). Normal distribution was tested with Shapiro Wilk test. Most nutrient values were not normally distributed, yet log-transformation did not improve normality. Next to absolute values, we calculated energy-adjusted nutrient values using the residual method by Willett et al. [35].

**Fig. 1:**
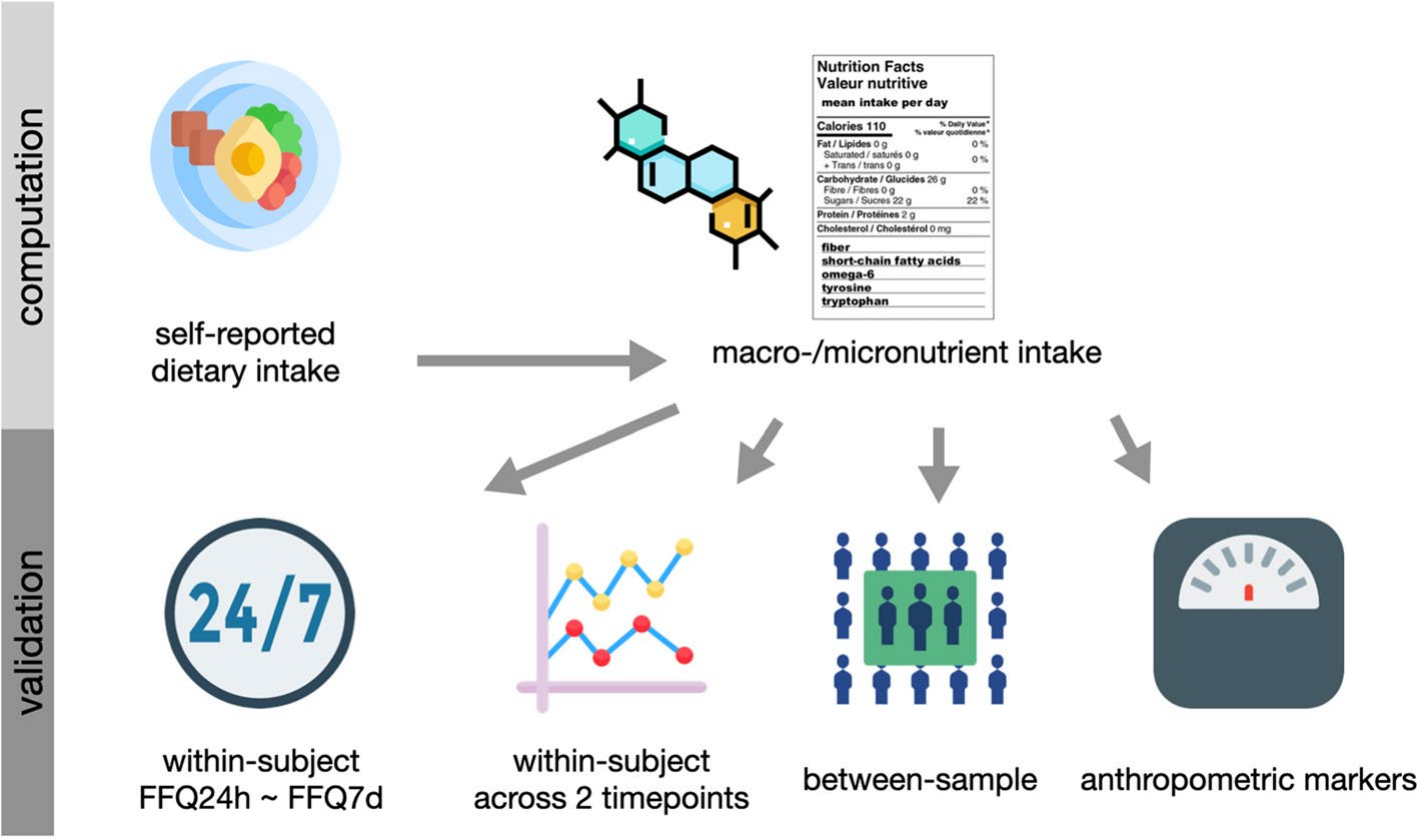
Schematic overview of nutrient intake computation and validation in this study

Intra-variability of two FFQs on different timescales, i.e. FFQ7d and FFQ24h, was assessed using Pearson’s correlation coefficients (after checking for normal distribution) with a significance level of α = 0.05 across all participants and both timepoints (Fig. 1). Moreover, reliability of nutrient scoring across two timepoints for identical nutrient outcomes was assessed using Intraclass Correlation Coefficient (ICC) with ICC() function in psych-package (version 2.1.9) for both FFQ 7 days and 24 h using two-way random, single measures for absolute agreement (ICC2) and guidelines for interpretation according to Koo and Li [36]. To test correlations between nutrient intake and biomarkers, Pearson’s correlation matrices were created using corrplot-package (version 0.90).

### Exploratory analysis

We used Pearson’s correlation to test whether main dietary intake variables (based on DEGS1-FFQ data) correlate with anthropometrics (BMI, body fat, WHR) and blood pressure as a measure of cardiovascular risk (Fig. 1). Further, linear mixed models (lme4-package, version 1.1.27.1.) were used to account for multiple datapoints from the same subject as well as for age, sex, and other confounding factors such as physical activity measured in MET-minutes / week based on IPAQ data [37].

## Results

### Computed nutrient intake

**Fig. 2:**
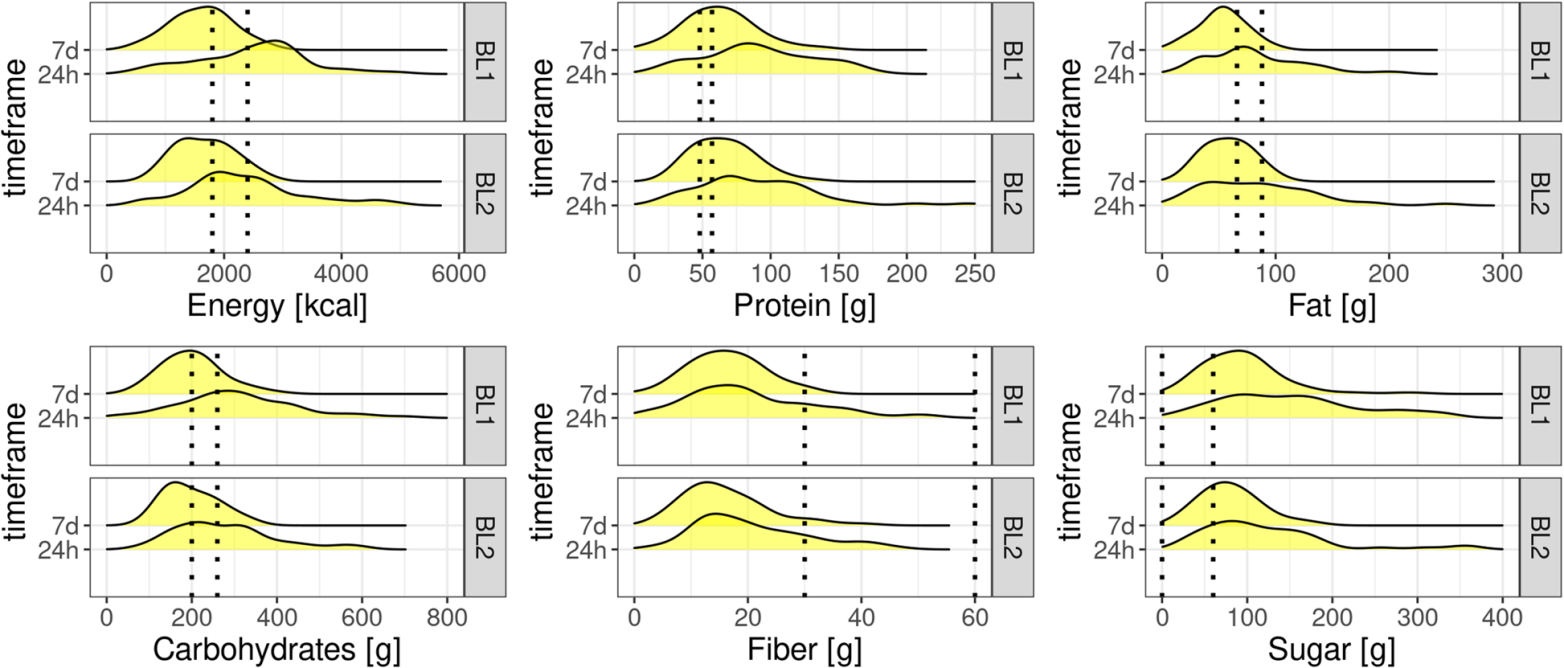
Frequency for computed macronutrients for FFQ 7 days and 24 h (*n* = 110 datapoints in total from first and second baseline). Dotted lines represent reference values (sources: “German Nutrition Counselling Network (DEBI)” for sugar, “German Society for Nutrition” for other nutrients)

Regarding food intake over the last 7 days as the sum of all 53 food group items, computed mean caloric intake matched reference values of 2000 kcal quite well, as well as protein (0.8 g/kg body weight/d based on German Society for Nutrition (DGE) guidelines) and fat intake (30% of overall energy intake recommended by DGE, for 2400 kcal 80–80 g/d) (Table 1, Fig. 2). Sugar intake in our sample was almost twice as high than the recommended 50 g/d (based on German Nutrition Counselling Network (DEBI)). Carbohydrate intake was on the lower recommended range (45–60% of overall energy intake based on European Food Safety Authority (EFSA) guidelines [38]) and fiber intake almost 50% lower than recommended by DGE (minimum of 30 g/d. Nutrient intake based on 7 days FFQ was overall higher in males than in females. Regarding the last 24 h, computed nutrient intake was on average higher than regarding the last 7 days. However, relative intake of nutrients reflected reference nutrient values (Table 2, Fig. 2), except for sugar which was 300% of the recommended intake and fiber which was again almost 50% lower than recommended. Although nutrient levels were mostly close to recommended levels on average, there was a large inter-individual variability for all nutrients for both 7 days and 24 h FFQ, but this was more pronounced for 24 h FFQ data (SI-Fig. 1 + 2). Computed nutrient intake from BL1 and BL2 only differed regarding energy-adjusted sugar intake (SI-Table 1). On the other hand, comparing FFQ24h and FFQ7d, energy intake was significantly higher for 24 h, but energy-adjusted nutrient intake did not differ (SI-Table 2).

**Table 1:** Nutrient intake descriptives based on FFQ 7 days (*n* = 59). F: female, M: male, BL1/BL2: baseline visits

FFQ 7 days by timepoint and gender				
	**BL1**		**BL2**	
	**F**	**M**	**F**	**M**
	**(*n* = 20)**	**(*n* = 39)**	**(*n* = 13)**	**(*n* = 36)**
**Energy [kcal]**				
Mean (SD)	1410 (424)	1730 (521)	1500 (467)	1740 (487)
Median [Min, Max]	1440 [501, 1930]	1810 [628, 2730]	1300 [919, 2350]	1770 [830, 2780]
**Protein [g]**				
Mean (SD)	50.0 (21.2)	70.4 (26.2)	58.6 (23.1)	68.1 (21.9)
Median [Min, Max]	47.0 [7.85, 86.5]	67.4 [16.7, 140]	47.5 [33.0, 106]	66.1 [27.0, 130]
**Fat [g]**				
Mean (SD)	43.3 (19.9)	58.7 (19.5)	50.4 (20.3)	60.9 (19.2)
Median [Min, Max]	47.7 [4.54, 71.8]	54.3 [17.6, 99.8]	41.3[24.0, 84.8]	63.3 [25.4, 99.3]
**Sugar [g]**				
Mean (SD)	97.8 (37.4)	92.2 (51.4)	86.0 (30.2)	78.8 (37.7)
Median [Min, Max]	98.0 [46.6, 223]	88.7 [25.9, 290]	78.7 [46.7, 160]	73.7 [19.1, 180]
**Carbohydrates [g]**				
Mean (SD)	184 (60.4)	210 (71.7)	182 (56.6)	204 (64.3)
Median [Min, Max]	182 [76.8, 360]	213 [80.3, 398]	181 [112, 297]	194 [91.0, 339]
**Fiber [g]**				
Mean (SD)	15.2 (6.45)	16.9 (6.18)	15.9 (9.32)	16.0 (6.78)
Median [Min, Max]	15.4 [1.54, 24.4]	15.5 [6.23, 30.5]	13.7 [6.34, 40.0]	15.2 [4.16, 32.7]
**Sat. FA [mg]**				
Mean (SD)	19,000 (9120)	26,000 (9920)	22,800 (9950)	26,800 (8380)
Median [Min, Max]	19,500 [2530, 31500]	24,600 [8150, 51800]	18,800 [8890, 43200]	26,700 [13100, 45100]
**Tyrosine [mg]**				
Mean (SD)	1810 (833)	2590 (1090)	2110 (859)	2440 (812)
Median [Min, Max]	1730 [228, 3200]	2360 [561, 5970]	1700 [1200, 3820]	2350 [1020, 4770]
**Tryptophan [mg]**				
Mean (SD)	592 (252)	836 (311)	696 (266)	810 (259)
Median [Min, Max]	578 [85.0, 1060]	834 [199, 1650]	575 [393, 1210]	790 [291, 1560]
**Omega-3 [mg]**				
Mean (SD)	1110 (782)	1230 (566)	1720 (1540)	1430 (710)
Median [Min, Max]	1050 [39.1, 3600]	1060 [529, 2670]	1210 [418, 6190]	1240 [342, 3000]
**Omega-6 [mg]**				
Mean (SD)	6910 (3710)	8320 (2860)	6400 (2320)	9060 (3690)
Median [Min, Max]	7030 [343, 14500]	7940 [2460, 14700]	6170 [3390, 10400]	9570 [3260, 17100]

**Table 2:** Nutrient intake descriptives based on FFQ 24 h (n = 55). F: female, M: male, BL1/BL2: baseline visits

FFQ 24 h by timepoint and gender				
	**BL1**		**BL2**	
	**F**	**M**	**F**	**M**
	**(*n* = 21)**	**(*n* = 34)**	**(*n* = 13)**	**(*n* = 35)**
**Energy [kcal]**				
Mean (SD)	2220 (1050)	2630 (862)	2010 (1070)	2490 (916)
Median [Min, Max]	2310 [421, 4110]	2770 [729, 4950]	1990 [597, 4530]	2440 [844, 4930]
**Protein [g]**				
Mean (SD)	83.7 (56.0)	101 (37.7)	74.7 (37.8)	95.4 (46.1)
Median [Min, Max]	76.1 [10.3, 259]	98.6 [27.6, 161]	65.5 [16.0, 147]	92.2 [28.4, 245]
**Fat [g]**				
Mean (SD)	69.8 (41.4)	89.0 (43.4)	71.5 (50.5)	89.1 (48.2)
Median [Min, Max]	68.9 [8.61, 167]	80.5 [32.3, 209]	51.6 [9.75, 188]	82.0 [13.4, 250]
**Sugar [g]**				
Mean (SD)	165 (121)	153 (76.6)	118 (85.8)	122 (73.8)
Median [Min, Max]	150 [12.4, 533]	145 [28.3, 334]	107 [27.5, 356]	99.8 [23.3, 357]
**Carbohydrates [g]**				
Mean (SD)	291 (165)	321 (119)	244 (127)	292 (116)
Median [Min, Max]	261 [19.9, 701]	317 [55.0, 618]	218 [76.4, 541]	272 [125, 598]
**Fiber [g]**				
Mean (SD)	22.2 (18.3)	21.1 (11.7)	17.6 (9.29)	21.9 (9.89)
Median [Min, Max]	17.8 [1.92, 88.1]	19.2 [2.10, 51.0]	15.2 [3.50, 37.9]	19.3 [6.23, 45.5]
**Sat. FA [mg]**				
Mean (SD)	29,600 (16,600)	41,000 (20,500)	32,500 (22,400)	39,700 (19,900)
Median [Min, Max]	30,500 [4930, 66400]	35,400 [14100, 96400]	23,500 [5010, 82400]	36,000 [7100, 101000]
**Tyrosine [mg]**				
Mean (SD)	2980 (2050)	3760 (1540)	2690 (1380)	3430 (1690)
Median [Min, Max]	2690 [306, 8990]	3630 [1050, 6160]	2190 [421, 5070]	3120 [869, 8910]
**Tryptophan [mg]**				
Mean (SD)	980 (661)	1220 (444)	893 (471)	1140 (569)
Median [Min, Max]	891 [121, 3130]	1200 [369, 1950]	745 [174, 1760]	1050 [289, 3010]
**Omega-3 [mg]**				
Mean (SD)	1850 (2180)	2020 (1820)	1890 (2000)	1820 (1190)
Median [Min, Max]	1330 [62.5, 8440]	1400 [77.9, 8860]	922 [106, 7220]	1650 [234, 4410]
**Omega-6 [mg]**				
Mean (SD)	11,300 (10,100)	11,300 (6660)	8290 (7500)	12,300 (8480)
Median [Min, Max]	8650 [518, 42600]	11,000 [1850, 31700]	5880 [1040, 29700]	9680 [1330, 37400]

### Reliability across two timepoints of assessment of nutrient intake

Test–retest reliability of dietary intake in 61 individuals was assessed with Pearson’s correlation and with intra-class correlation coefficient (ICC) to assess the agreement between two assessments with the same instrument for all energy-adjusted nutrient values.

For FFQ7d, there was moderate absolute agreement between the two data assessments, for all macronutrients (Pearson’s rho_min_ = 0.33, rho_max_ = 0.64, kappa_all_ ≥ 0.36, kappa_max_ = 0.62), with highest agreement for fiber and lowest for fat intake. For FFQ24h, agreement between the two timepoints was poor to moderate overall (Pearson’s rho_min_ = 0.13, rho_max_ = 0.45, kappa_all_ ≥ 0.11, kappa_max_ = 0.46, Table 3), and also highest for fiber but lowest for sugar intake.

**Table 3:** Test–retest reliability of energy-adjusted nutrient values between two assessment timepoints for both, FFQ7d and FFQ24h

	FFQ7d BL1 ~ BL2		FFQ24h BL1 ~ BL2	
Energy-adjusted nutrient value	Pearson’s rho	ICC kappa (ICC2) [lower bound, upper bound]	Pearson’s rho	ICC kappa (ICC2) [lower bound, upper bound]
Protein	0.50	0.50 [0.32, 0.64]	0.30	0.33 [0.13, 0.51]
Fat	0.33	0.36 [0.16, 0.53]	0.38	0.37 [0.17, 0.53]
Carb	0.54	0.50 [0.33, 0.65]	0.24	0.24 [0.03, 0.42]
Fiber	0.64	0.62 [0.47, 0.73]	0.45	0.46 [0.28, 0.61]
Sugar	0.61	0.62 [0.42, 0.75]	0.13	0.11 [-0.08, 0.30]

### Reliability between 7 days and 24 h FFQ nutrient intake

In general higher nutrient scores for 7d correlated with higher nutrient scores for 24 h, respectively, resembling that individuals who reported high nutrient intake on the FFQ7d also reported high nutrient intake on the FFQ24h. Intra-individual variability between 7 days and last 24  for all energy-adjusted nutrients of interest was moderate (Pearson’s rho_all_ ≥ 0.54, rho_max_ = 0.62, kappa_all_ ≥ 0.39, kappa_max_ = 0.50, Table 4).

**Table 4:** Test–retest reliability of energy-adjusted nutrient values between 7 days and 24 h FFQ

	FFQ7d ~ FFQ24h	
Energy-adjusted nutrient value	Pearson’s rho	ICC kappa (ICC2) [lower bound, upper bound]
Protein	0.57	0.46 [0.33, 0.57]
Fat	0.58	0.40 [0.27, 0.52]
Carb	0.55	0.39 [0.25, 0.51]
Fiber	0.54	0.41 [0.28, 0.53]
Sugar	0.62	0.50 [0.37, 0.60]

### Extension of nutrient scoring by two independent samples

An additional cross-sectional sample consisting of German university cafeteria visitors showed similar nutrient values and deviations from the reference values (Table 5). Calorie intake was lower than expected reference values, protein and fat matched reference ranges well, sugar intake was 60% higher than recommended. Carbohydrate was slightly lower and fiber intake 30% lower than recommended.

**Table 5:** Computed nutrient intake for a cross-sectional sample of university cafeteria visitors for FFQ 14 days (*n* = 134)

FFQ 14 days nutrient intake by gender				
	**female**	**male**	**diverse**	**overall**
	**(*n* = 79)**	**(*n* = 43)**	**(*n* = 1)**	**(*n* = 134 incl. 11 NA’s for gender)**
**BMI (kg/m**^**2**^**)**				
Mean (SD)	22.6 (3.40)	22.5 (2.37)	22.0 (NA)	22.5 (3.05)
Median [Min, Max]	22.0 [18.0, 40.6]	22.7 [17.5, 27.2]	22.0 [22.0, 22.0]	22.3 [17.5, 40.6]
**Energy [kcal]**				
Mean (SD)	1440 (571)	1640 (595)	1510 (NA)	1500 (576)
Median [Min, Max]	1360 [350, 3070]	1530 [758, 3040]	1510 [1510, 1510]	1400 [350, 3070]
**Protein [g]**				
Mean (SD)	52.0 (22.1)	68.5 (31.4)	59.0 (NA)	57.0 (26.9)
Median [Min, Max]	48.1 [10.9, 161]	58.3 [28.2, 154]	59.0 [59.0, 59.0]	51.2 [10.9, 161]
**Fat [g]**				
Mean (SD)	49.9 (35.8)	53.1 (23.1)	35.7 (NA)	49.6 (30.9)
Median [Min, Max]	40.0 [12.7, 198]	49.2 [21.6, 115]	35.7 [35.7, 35.7]	40.4 [12.7, 198]
**Sugar [g]**				
Mean (SD)	89.5 (48.4)	80.3 (58.0)	57.2 (NA)	84.4 (50.5)
Median [Min, Max]	85.4 [18.2, 341]	69.5 [23.6, 360]	57.2 [57.2, 57.2]	77.0 [18.2, 360]
**Carbohydrates [g]**				
Mean (SD)	182 (68.5)	198 (78.5)	175 (NA)	187 (71.6)
Median [Min, Max]	177 [44.2, 404]	197 [93.7, 472]	175 [175, 175]	185 [44.2, 472]
**Fiber [g]**				
Mean (SD)	19.1 (10.1)	18.2 (9.97)	22.1 (NA)	18.7 (9.83)
Median [Min, Max]	17.6 [4.11, 60.5]	15.1 [6.08, 46.9]	22.1 [22.1, 22.1]	17.0 [4.11, 60.5]
**Sat. FA [mg]**				
Mean (SD)	22400 (17100)	22800 (11400)	12200 (NA)	21900 (14800)
Median [Min, Max]	18000 [4380, 97900]	20800 [8060, 56600]	12200 [12200, 12200]	17500 [4380, 97900]
**Tyrosine [mg]**				
Mean (SD)	1880 (882)	2490 (1270)	2170 (NA)	2060 (1080)
Median [Min, Max]	1760 [370, 6510]	2080 [949, 6780]	2170 [2170, 2170]	1830 [370, 6780]
**Tryptophan [mg]**				
Mean (SD)	606 (255)	817 (380)	721 (NA)	672 (320)
Median [Min, Max]	574 [120, 1840]	700 [313, 1820]	721 [721, 721]	597 [120, 1840]
**Omega-3 [mg]**				
Mean (SD)	1030 (820)	3510 (13,500)	1220 (NA)	2040 (8100)
Median [Min, Max]	708 [332, 30400]	1410 [388, 89900]	1220 [1220, 1220]	874 [213, 89900]
**Omega-6 [mg]**				
Mean (SD)	7810 (7060)	8300 (3310)	7280 (NA)	7790 (5780)
Median [Min, Max]	6040 [2380, 41300]	7660 [1840, 16400]	7280 [7280, 7280]	6610 [1820, 41300]

From another cross-sectional sample including men only (*n* = 76) with a mean BMI of 23.6 ± 2.7 kg/m^2^ (range: 18.6–36.4 kg/m^2^) and mean age of 26.6 ± 4.4 years (range: 18–40 y), we calculated nutrient values of a FFQ recall period of four weeks (Table 6). The sample was grouped into high and low dietary fat and sugar consumers (HFS vs. LFS) based on DFS scores. Overall, calorie intake, protein, and fat matched reference ranges well. Fiber was slightly lower than recommended (< 30 g) for both groups. Groups differed significantly in overall calorie intake and the HFS group presented significantly higher levels of HbA1c, a long-term marker of glucose metabolism. The statistical comparison of the residuals of the energy-adjusted nutrients revealed a significant difference between protein and fiber intake with higher intake in HFS vs. LFS group, but not between fat, carbohydrates and sugar intake. Notably, both groups surpassed recommended intake of sugar (about 1.5–2.5 × higher than recommended), yet the HFS group did so by far.

**Table 6:** Computed nutrient intake for a cross-sectional sample of adult men for FFQ 28 days

FFQ 28 days nutrient intake grouped by DFS score			
	**HFS**	**LFS**	***p*-value**
	**(*n* = 35)**	**(*n* = 41)**	
**Diet**			
OMN	32 (91.4%)	27 (65.9%)	
VEG	3 (8.6%)	14 (34.1%)	
**HbA1c (mmol/mol)**			
N-Miss	1	0	
Mean (SD)	33.471 (2.549)	32.014 (3.047)	**0.030**
Median [Min, Max]	33.38 [28.3, 37.9]	32.24 [22.8, 37.2]	
**Energy [kcal]**			
Mean (SD)	2540 (654)	1710 (694)	** < 0.001**
Median [Min, Max]	2430 [1360, 4050]	1620 [664, 3540]	
**Protein [g]**			
Mean (SD)	95.4 (26.3)	75.6 (42.5)	**0.002**
Median [Min, Max]	92.9 [43.2, 156]	62.7 [23.0, 213]	
**Fat [g]**			0.822
Mean (SD)	92.4 (33.0)	59.4 (27.8)	
Median [Min, Max]	87.7 [43.6, 196]	52.6 [14.5, 126]	
**Carbohydrates [g]**			
Mean (SD)	308 (87.1)	204 (87.3)	0.428
Median [Min, Max]	294 [189, 584]	201 [46.2, 461]	
**Fiber [g]**			
Mean (SD)	27.8 (10.4)	25.5 (13.0)	**0.020**
Median [Min, Max]	25.0 [11.1, 56.4]	22.2 [8.69, 62.1]	
**Sugar [g]**			
Mean (SD)	132 (61.2)	85.9 (39.4)	0.822
Median [Min, Max]	115 [47.5, 343]	82.6 [25.2, 171]	

*HFS* High Fat and Sugar Group, *LFS* Low Fat and Sugar Group, *OMN* omnivorous, *VEG* vegetarian. *P*-values are indicated for standard 2-sample t.test for numeric variables and for chi-squared tests of independence for categorical variables. Statistical comparison of the nutrients (except energy) was conducted with the residuals of the energy-adjusted values

Regarding differences for dietary adherence groups (omnivorous *n* = 59, vegetarian *n* = 17), most vegetarians were in the LFS group (82%) (SI-Table 3). Compared to omnivorous dieters, vegetarians reported significantly higher fiber intake (*p* = 0.009). The diet groups did not differ in any other macronutrient value or in HbA1c.

**Fig. 3:**
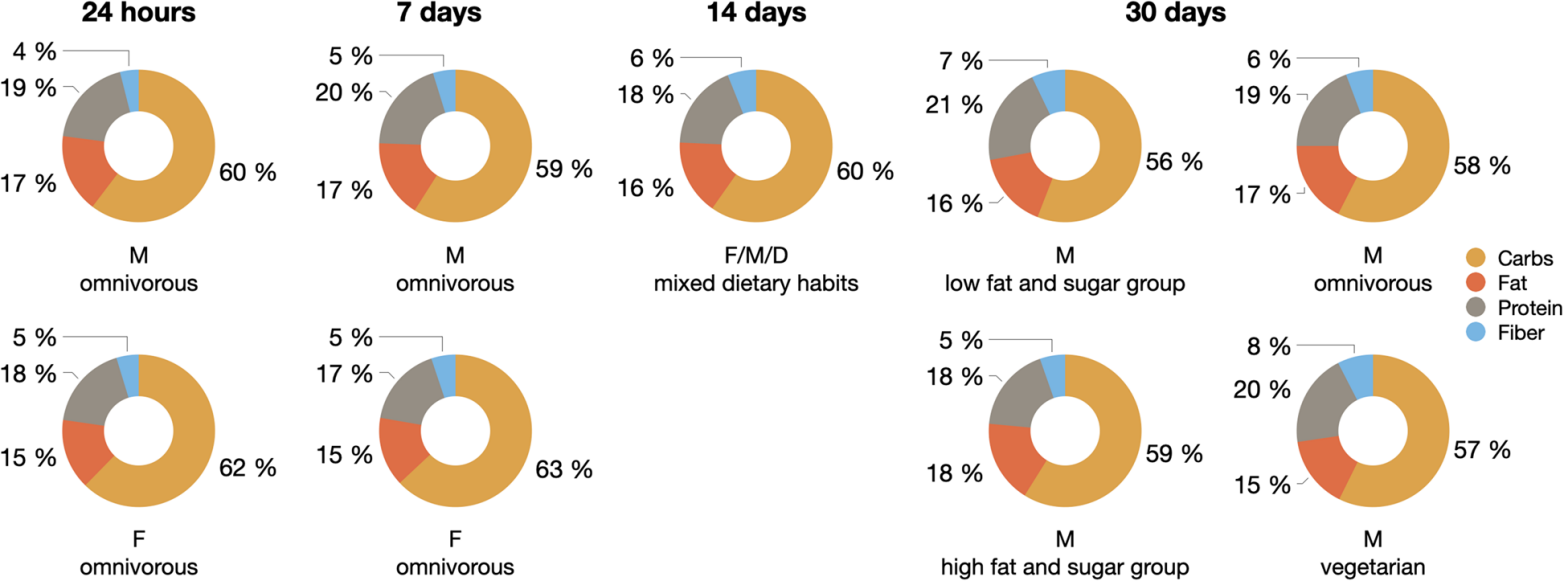
Pie charts for proportions of computed nutrient intakes per time frame of questionnaire per dataset in percent for carbohydrates, fat, protein and fiber intake. 24 h and 7 days are based on data from the first baseline of the GUT-BRAIN dataset. Calorie intake was not considered here. Abbreviations: F: female, M: male, D: diverse

Overall macronutrient composition of nutrient intake was comparable for FFQ data from different time periods (24 h, 7d, 14d, 30d) and consisted across samples of 56–63% carbohydrates, 15–18% fat, 17–21% protein, 4–8% fiber (Fig. 3).

### Correlation of computed nutrient intake with anthropometric markers

**Fig. 4:**
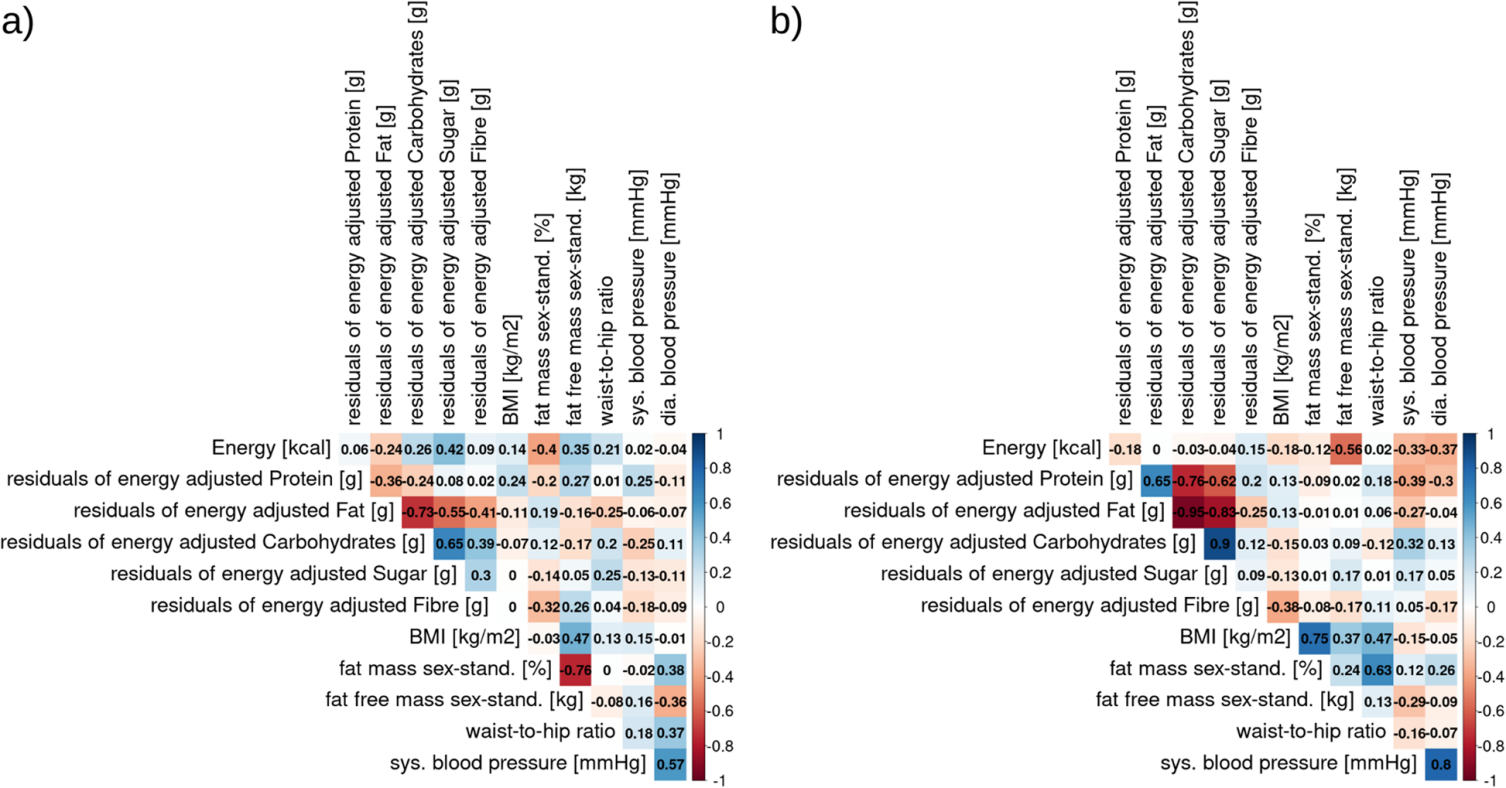
Correlations (Pearson’s correlation coefficient) of nutrient-related markers and anthropometric markers for BL1 timepoint only for a) males only and b) females only

We tested if computed nutrient intake was related to anthropometric measures in the overweight, omnivorous, main sample (for sample descriptives see SI Fig. 3 + 4). Firstly, sex-standardized body fat mass was highly anti-correlated with sex-standardized fat-free mass in males (r = -0.76), yet the inverse was true for females (r = 0.24). In addition, only females showed high accordance of BMI and sex-standardized body fat mass (r = 0.75, Fig. 4a-b). Due to those differences in anthropometrics by sex, we considered sex-stratified analyses in further steps.

Regarding computed nutrient intake, residuals of energy-adjusted fat intake was moderately to strongly anti-correlated with intake of carbohydrates, sugar and fiber in males (r = -0.73, r = -0.55, and -0.41 respectively, Fig. 4a). In females, the anti-correlation of energy-adjusted fat intake with carbohydrates and sugar intake was even stronger than in males (r = -0.95 and r = -0.83 respectively, Fig. 4b). In addition, females' eneryg-adjusted protein intake strongly anti-correlated with carbohydrate and sugar intake (r = -0.76 and r = -0.62 respectively), but showed a strong positive association with fat intake (r = 0.65, Fig. 4b). Females showed moderate anti-correlation between BMI and fiber intake (r = -0.38), but no further moderate or high correlation was evident between nutrient intake and BMI or WHR in females or males. In males, energy intake was moderately anti-correlated with sex-standardized body fat mass (r = -0.40) and moderately positively correlated with fat free mass (r = 0.35), whereas in females, we found a moderate anti-correlation of energy intake with fat free mass (r=-0.56) and a low anti-correlation with fat mass (r = -0.12). In females, systolic and diastolic blood pressure was found to be moderately anti-correlated with energy intake and energy-adjusted protein intake, while systolic blood pressure moderately correlated with energy-adjusted carbohydrate intake and weakly anti-correlated with energy-adjusted fat intake. Only weak correlations with systolic blood pressure (positive with protein, negative with carbohydrate intake) were found in males. 

**Fig. 5:**
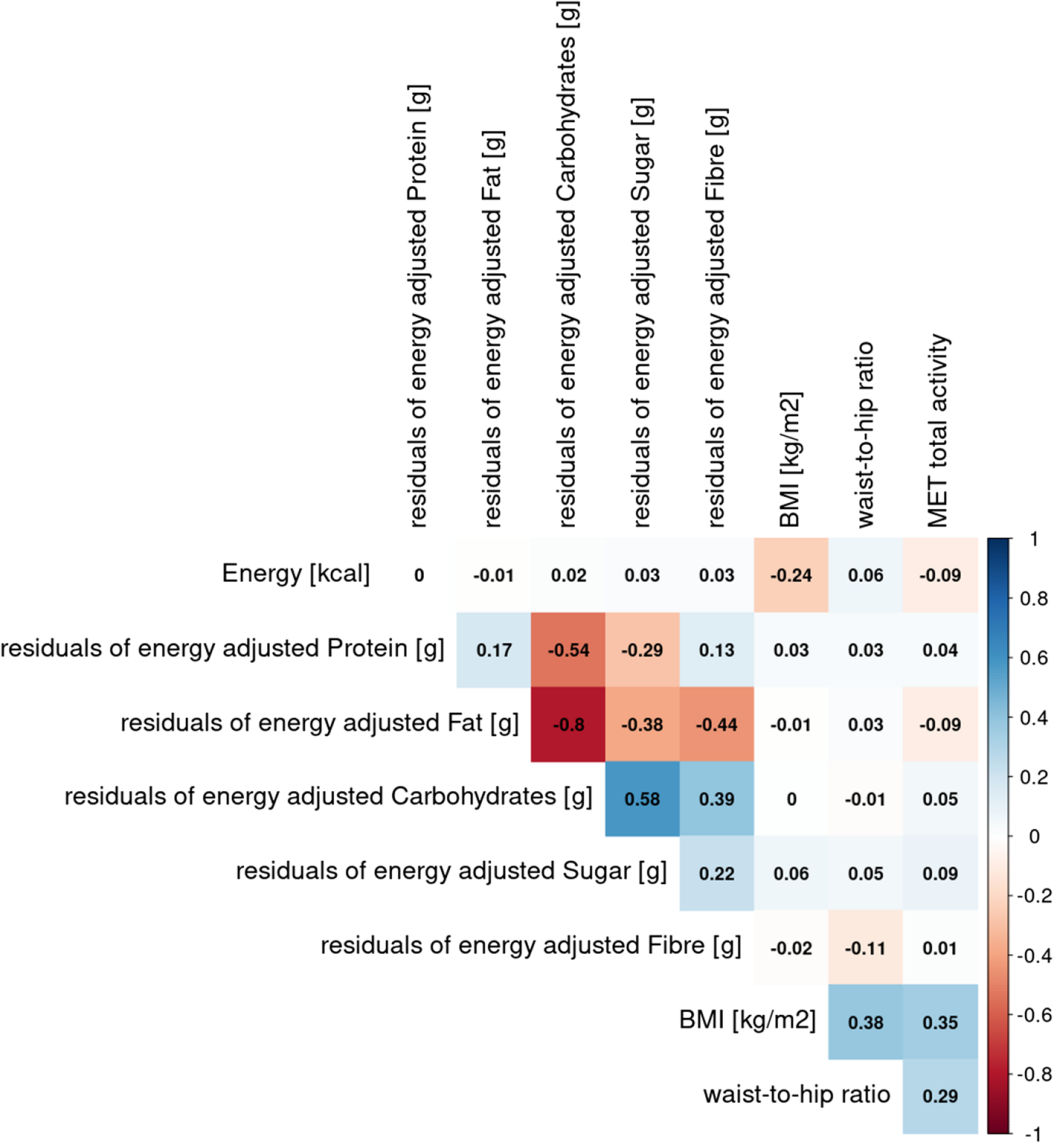
Correlations (Pearson’s correlation coefficient) between computed nutrient intake and anthropometric markers for a BMI range of 18.6–36.4 kg/m2 of male and female participants (GUT-BRAIN + GREADT)

When merging samples and looking at the whole weight range from normal-weight to obese (*n* = 187, BMI: 18.6–36.4 kg/m^2^ M ± SD: 25.9 ± 2.8; WHR: 0.65–0.98, M ± SD: 0.81 ± 0.05; data from GUT-BRAIN and GREADT), negative correlations of protein and fat intake with carbohydrates intake remained high. In this sample with a broad BMI range, associations of nutrient intake with BMI and WHR were not considerable except for a weak negative correlation of BMI with energy intake (Fig. 5). However, linear mixed models adjusted for age, sex, and physical activity (MET-minutes per week) with subject as random factor, did not indicate significant predictions of BMI or WHR by residuals of energy-adjusted nutrient intake (full-null model comparisons: all p > 0.05, see SI-Table 4 & SI-Table 5). In the overweight, main sample, higher fat intake (full null model comparison: *p* = 0.03, SI-Table 4) and lower fiber intake (full null model comparison: *p* = 0.03) predicted higher BMI. Higher WHR on the other hand was only significantly related to higher energy intake (full null model comparison: *p* < 0.04, SI-Table 5). When extending the analysis with linear mixed models to the wider BMI range, predictions did not remain significant.

## Discussion

We translated self-reported food intake (using the commonly used German DEGS1-FFQ) of different samples into detailed nutrient intake per day for various macro- and micronutrients and dietary components. Additionally, we assessed test–retest and between-timeframe reliability, and made the scoring scripts openly available. Although we assume a large proportion of under- and overreporting on the individual’s level, the nutrient values mostly met recommended reference intake on average, except for higher sugar and lower fiber intake then recommended. This is in line with studies across different European countries. These studies showed that sugar intake can make up to 115 g/d or 20% of overall energy intake [39] and that fiber intake was only 14–21 g/d on average, thereby not reaching recommended levels [40]. As expected, males consumed higher amounts of nutrients than females, and differences in nutrient intake were present across groups of different dietary adherence or eating habits, such as between omnivorous and vegetarian dieters or groups with high and low fat and sugar intake.

Assessing reliability over two timepoints within one month, we found moderate agreement for FFQ7d (Pearson’s rho_min_ = 0.33, rho_max_ = 0.64, kappa_all_ ≥ 0.36, kappa_max_ = 0.62), similar to a previous validation study based on food groups [41], with highest reliability for fiber and lowest for fat. For FFQ24h, reliability was lower (Pearson’s rho_min_ = 0.13, rho_max_ = 0.45, kappa_all_ ≥ 0.11, kappa_max_ = 0.45), with highest reliability for fiber but lowest for sugar. Indeed, the difference between FFQ7d and FFQ24h could reflect individual variance in eating habits that are more consistent over a time course of one week than on single days. The FFQ24h was often filled out during weekend days, when actual food intake might be more variable than on regular weekdays. To achieve highest correlation, the administration of four 24hR has been recommended [42]. Therefore, the use of only two FFQ24h in our study might have led to underpowered results due to overrepresentation of weekend days and therefore lower consistency. However, when we compared energy-adjusted nutrient values, only reported sugar intake turned out to differ significantly between the two timepoints. In summary, FFQs with longer time periods (e.g. 7 days or more) have higher reliability and should be used for assessing dietary habits or intake. Additionally, accounting for energy intake with the residual method [35] guarantees better comparability of the nutrient values. FFQs relating to shorter time periods (e.g. 24 h) may be especially helpful to assess diet as a confounder variable, e.g. for microbial sampling.

Reliability between last 7 days and 24 h FFQ was moderate for all nutrients of interest (Pearson's rho_all_ ≥ 0.54, rho_max_ =0.62, kappa_all_ ≥ 0.39, kappa_max_ = 0.50). In the validation study of the (original) DEGS1-FFQ on the food group level, reliability was assessed between 28 days FFQ and two 24 h recalls by phone and ranged from low to good across 53 food items. Most validation studies use multiple 24 h recalls per participant to assess dietary intake of different days. This way, day-to-day variations of food consumption can be recorded [43] and the imprecision of FFQs at the individual level can be adjusted [42, 44]. In comparison to the item-wise correlation, we computed reliability for nutrient intake summed over all food groups for 7 days and 24 h assessed with the same online tool. Comparing energy adjusted nutrient values between FFQ7d and FFQ24h, differences did not present significant. The overall better performance of our nutrient scoring compared to the original food group scoring can be attributed to the reduction of outcome nutrient variables (six macronutrient values vs. 53 food groups). Additionally, nutrient intake patterns compared to single food item intake might have a higher consistency. Not only the reduction of outcome variables but also because we additionally considerate specific variations of certain food groups in our nutrient calculation, we improved the validity of the FFQ scoring. These specific variations, if left unconsidered as in the original FFQ scoring considerably define the nutrient values of the respective food group (e.g. fried vs non-fried meat/fish coffee/tea with or without sugar). For exemplary calculations of energy value variations see SI-Table 6. The nutrient scoring of the DEGS1-FFQ which we provide can thus be considered valid in terms of assessing relatively similar nutrients and food groups when reporting 7 days or to a lesser degree 24 h.

We further evaluated the sensibility of the computed nutrient data and extended our nutrient scoring for DEGS1-FFQ data to two additional samples. The first additional sample consisted of mixed genders and omnivores only (as in the main sample) and showed comparable nutrient intake even though the BMI range was wider than the main sample’s. The second additional, male-only sample with vegetarians and omnivores divided into groups with high and low fat and sugar intake (HFS / LFS) showed not only a significant difference in HbA1c, a long-term glucose marker, reflecting a higher risk for diabetes development, coronary heart disease or stroke in the long-term [45], but also in energy intake (HFS > LFS). This indicated that our nutrient results reflects actual eating habits. Absolute fat and sugar intake was higher in the HFS group compared to the LFS group, however, after adjusting for energy intake, this difference was not significant. On the other hand, energy-adjusted protein and fiber intake differed significantly between DFS groups. This result followed the expectation that higher energy intake through higher fat and sugar intake in the HFS group must be accompanied by a proportionally lower protein and fiber intake when nutrients are adjusted for energy intake.

Associations of anthropometrics with energy-adjusted nutrient intake showed mixed results. In the omnivorous, overweight, main sample, energy-adjusted protein and fat intake were moderately to highly anti-correlated with intake of carbohydrates. This emphasized that, independent of energy intake, carbohydrate intake proportionally decreased when fat and protein intake increased. As expected, higher energy-adjusted fiber intake across 7 days was moderately linked to lower BMI in females (but not males) and to percental lower fat mass in males (but not females). This points towards healthier diets high in fiber intake relating to lower weight / percental fat mass in that sample. A link between fiber and lower weight has been shown before for diets restricted in animal-based foods [46] and systematically reviewed for whole-grain and fiber-rich foods [8]. For the other energy-adjusted nutrients, no evident link to BMI was found. In the main sample, WHR was not considerably linked to any of the energy-adjusted nutrient values. Blood pressure (BP) though presented with mixed associations; in females, systolic and diastolic BP was found to be moderately anti-correlated with energy intake and energy-adjusted protein intake, while systolic BP moderately correlated with energy-adjusted carbohydrate intake. In males, however, only weak correlations were found. The link between high-protein diets and lower BP in females, has some to no evidence from meta-analyses [47, 48], while the link between higher energy intake and lower BP remains to be investigated. Regarding the correlation of carbohydrate intake and BP, evidence is weak but pointing towards lower BP in low-carb diets [49]. When looking at the larger merged sample spanning from normal-weight to obese, all links between energy-adjusted nutrients and BMI as well as WHR presented as insignificant, except for a weak negative correlation of BMI with energy intake. However, when fitting linear models accounting for interdependency of datapoints and for age, sex and physical activity (MET-minutes per week), neither BMI nor WHR were predicted by any nutrient value. 

Thanks to our nutrient calculations and the concomitant option to adjust nutrient values for energy intake, we recommend to use linear mixed models for statistical comparison. Linear mixed models offer the possibility to adjust for sex, age and physical activity as well as to account for interdependency of datapoints. Especially between gender groups, previous studies report different results comparing food intake and anthropometric markers. For example, whole-grain intake was associated with lower BMI for both sexes, yet fiber in particular was inversely correlated with BMI only in men, not in women [50, 51] and likewise with immune function [52]. Other studies [53, 54] showed that fiber intake was not different between males and females. Nevertheless, proposed mechanisms of fiber intake in women may be metabolic benefits, i.e. reduced lipids in the blood, mediated by estradiol levels [55] and even blunted hormonal signaling during the reproductive cycle [56]. The picture on sex-specific associations of fiber intake on anthropometrics seems rather inconclusive and more studies are needed to disentangle sex-specific effects of fiber intake on metabolic, immune or reproductive markers. Until further clarification, we therefore recommend accounting for sex in statistical analyses.

A strength of our analysis is the pooling of data from two human studies with deeply phenotyped samples. These samples were selected based on research questions focusing on eating habits and merging them resulted in a large dataset with 187 datapoints. 

We encourage researchers to regard calculated nutrient intake as a putative measure of interest to be extracted from FFQ data. Such calculation pipelines are rarely if at all available. Therefore we publish all scripts open access and open code. Overall, we propose FFQs along with automated nutrient scoring as a powerful tool to assess dietary intake. Our automated pipeline may contribute to developing nutrient scoring further and to advance nutrition sciences. In which context fiber intake may be a powerful tool for weight management and dietetic treatments as proposed before [57, 58] remains to be investigated further. Interestingly, the link between lower BMI to higher fiber intake, we only found in the female, overweight and healthy sample offers valuable information but opens the question of gender-specific effects. Dietary intake, in particular high fiber diets, have a large potential in preventing obesity-related states and comorbidities on a societal level [8, 59]. We suggest to increase educational efforts on fiber content of foods (as it is oftentimes not printed on food packaging, or available in experimental datasets, e.g. Food-pics database [60]) and to ameliorate policy making in the food sector (public and private) [61] and nutrition communication [62] to enhance fiber-rich diets and food items.

Overall, nutritional epidemiology will benefit from more advanced nutrient assessment and future studies which reveal more insights on the impact of nutrient intake. Thereby, these studies would provide more reliable and comparable evidence to better inform public policy-making in the long-term.

## Limitations

Firstly, all data is based on self-reported questionnaires only, and more reliable objective measures for dietary intake such as doubly labelled water [63] or urine nitrogen [64] were not implemented. Also due to inherent structure of the FFQ used, imprecision in the results might remain. For instance, only 53 food items are covered in the DEGS1, which means that a variety of different food items is not taken into account leading to gaps in data acquisition (e.g. legumes/ soy products,…). Another inaccuracy might stem from the fact that only certain FFQ questions are accompanied by a visual prompt, such as a picture of the portion size of a certain food (as provided by the Robert Koch Institute). As a result, the lack of a benchmark when estimating food intake might have led to deviations in the assessment.

Secondly, large ranges of values were present for some nutrients. This can be attributed to occasional over- or underreporting, which is why we decided to exclude energy intake related outliers. This shows the variability and limitations of self-reported FFQ data at the nutrient level and the need for data curation strategies. These data curation strategies should depend on the research question and include definitions for implausible data entries by consensus decision of different raters.

Thirdly, as the main sample is partly from a dietary intervention study, characteristics of this sample may reflect a selection bias in favour of omnivorous eaters with some awareness of the study goals to influence eating behaviour. Yet, we cross-validated data from the main sample with two other samples, albeit also from studies focusing on eating behaviour. Overall, self-reported dietary data is never blinded and neutral, since participants may reflect on social desirability and therefore report in a biased way. Nevertheless, self-reported FFQ data in combination with recalls are a valid tool to assess nutrient intake [42] and relative (energy adjusted) nutrient intake can be validly compared. Also, although each participant came in on same day of the week, there may be a bias in FFQ24h data due to reported dietary intake referring to Tuesdays (33%) and weekend days (67%) disproportionately. Yet, energy-adjusted nutrient intake did not differ significantly between FFQ7d and FFQ24h.

## Conclusions

Our newly developed nutrient scoring allows to extract specific nutrient information of interest that can be further used to address specific research questions and to reduce dimensionality of FFQ outcomes, thus improving comparability for (self-reported) nutrient intake across studies. Reliability of computed nutrient values is similar to previously reported dietary intake for 24 h and 7 days reports. However, differences between questionnaire timeframes and assessment days present insignificant when adjusting nutrient values for energy intake. We believe that by making the scripts and descriptives for nutrient scoring available, we can provide the nutrition research community with more precise proxies for dietary intake, especially with respect to the German DEGS1-FFQ, but also by allowing to adopt the openly shared methodology to other questionnaires.

## Supplementary Information

**Additional file 1:SI-Figure 1.** Computed nutrient values plotted across two timepoints for FFQ 7 days. Transparent lines are colour-coded by the individual. **SI-Figure 2.** Computed nutrient values plotted across two timepoints for FFQ 24 hours. Individual lines are colour-coded by individual. **SI-Figure 3.** Distribution of anthropometric markers by sex. BL1 measures only for 59 individuals. **SI-Figure4.** Distribution of nutrient values by sex. BL1 measures only for 59 individuals. **SI-Table 1.** Computed, energy adjusted nutrient intake for across-sectional sample of adult women and men from two assessment days. *P*-values are indicated for standard 2-sample t.test. **SI-Table 2.** Computed, energy adjusted nutrient intake for a cross-sectional sample of adult women and men for FFQ 24 hours and FFQ 7 days. *P*-values are indicated for standard 2-sample t.test. **SI-Table 3.** Computed nutrient intake for a cross-sectional sample of adult men for FFQ 28 days. HFS = High Fat and Sugar Group, LFS = Low Fat and Sugar Group, OMN = omnivorous, VEG = vegetarian. Statistical comparison of the nutrients (except energy) was conducted with the residuals of the energy-adjusted values. *P*-values are indicated for standard 2-sample t.test. **SI-Table  4.** Results of linear mixed models for normal-weight to obese sample (n=187, BMI: 18.6-36.4 kg/m2 M±SD: 25.9±2.8; GUT-BRAIN and GREADT). Prediction of BMI by residuals of energy-adjusted nutrient values. General equations: H0: BMI ~ sex + age + MET total activity + (1/subject) H1: BMI ~ nutrient value + sex + age + MET total activity + (1/subject) Abbreviations: AIC: Akaike Information criterion; BIC: Bayesian information criterion. **SI-Table  5.** Results of linear mixed models for normal-weight to obese sample (n=187, WHR: 0.65-0.98, M±SD: 0.81±0.05; GUT-BRAIN and GREADT). Prediction of WHR by residuals of energy-adjusted nutrient values. General equations: H0: WHR ~ sex + age + MET total activity + (1/subject) H1: WHR ~ nutrient value + sex + age + MET total activity + (1/subject) Abbreviations: AIC: Akaike Information criterion; BIC: Bayesian information criterion. **SI-Table 6**. Computed energy value for two exemplary food items and their respective food subgroups. NB: Food items “Chicken” and its different preparation types and “Coffee” with different amounts of sugar. Last column emphasizes the difference in energy value if the additional information obtained by food subgroup questions was disregarded in the nutrient calculation as it is disregarded in the original scoring.

## Data Availability

All scripts are available here: https://gitlab.gwdg.de/omega-lab/ffq-nutrient-scoring/
