# Supplementary material for: Nutrient scoring for the DEGS1-FFQ – from food intake to nutrient intake
Source: BMC Nutr. 2023 Jan 13;9:12. doi: 10.1186/s40795-022-00636-2 (PMC9837986; doi:10.1186/s40795-022-00636-2)
Supplement: Supplementary file 1 — Additional file 1:SI-Figure 1. Computed nutrient values plotted across two timepoints for FFQ 7 days. Transparent lines are colour-coded by the individual. SI-Figure 2. Computed nutrient values plotted across two timepoints for FFQ 24 hours. Individual lines are colour-coded by individual. SI-Figure 3. Distribution of anthropometric markers by sex. BL1 measures only for 59 individuals. SI-Figure4. Distribution of nutrient values by sex. BL1 measures only for 59 individuals. SI-Table 1. Computed, energy adjusted nutrient intake for across-sectional sample of adult women and men from two assessment days. P-values are indicated for standard 2-sample t.test. SI-Table 2. Computed, energy adjusted nutrient intake for a cross-sectional sample of adult women and men for FFQ 24 hours and FFQ 7 days. P-values are indicated for standard 2-sample t.test. SI-Table 3. Computed nutrient intake for a cross-sectional sample of adult men for FFQ 28 days. HFS = High Fat and Sugar Group, LFS = Low Fat and Sugar Group, OMN = omnivorous, VEG = vegetarian. Statistical comparison of the nutrients (except energy) was conducted with the residuals of the energy-adjusted values. P-values are indicated for standard 2-sample t.test. SI-Table 4. Results of linear mixed models for normal-weight to obese sample (n=187, BMI: 18.6-36.4 kg/m2 M±SD: 25.9±2.8; GUT-BRAIN and GREADT). Prediction of BMI by residuals of energy-adjusted nutrient values. General equations: H0: BMI ~ sex + age + MET total activity + (1/subject) H1: BMI ~ nutrient value + sex + age + MET total activity + (1/subject) Abbreviations: AIC: Akaike Information criterion; BIC: Bayesian information criterion. SI-Table 5. Results of linear mixed models for normal-weight to obese sample (n=187, WHR: 0.65-0.98, M±SD: 0.81±0.05; GUT-BRAIN and GREADT). Prediction of WHR by residuals of energy-adjusted nutrient values. General equations: H0: WHR ~ sex + age + MET total activity + (1/subject) H1: WHR ~ nutrient value + sex + age + MET total a [file 40795_2022_636_MOESM1_ESM.docx]

### Supplementary Information


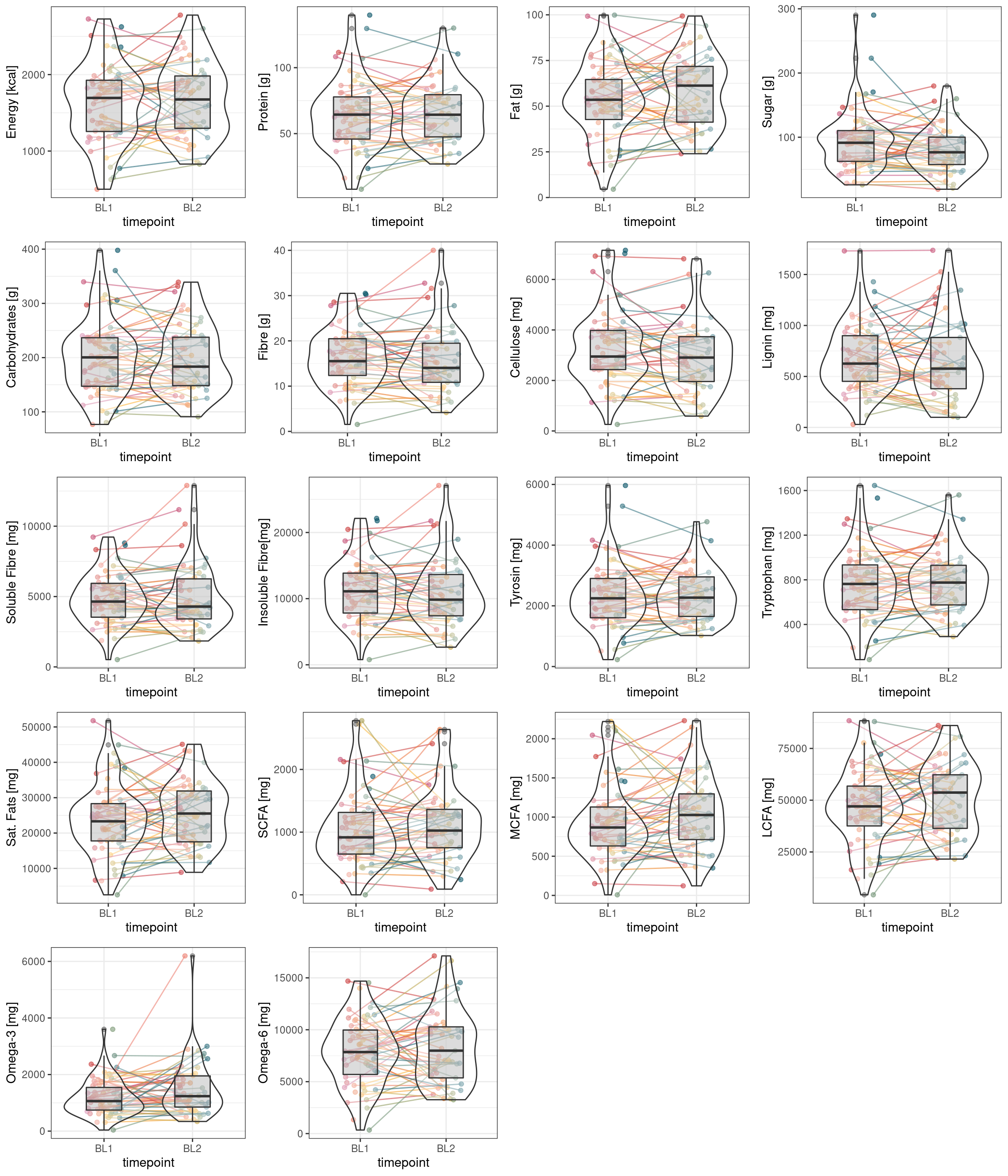


*SI-Figure 1: Computed nutrient values plotted across two timepoints for FFQ 7 days. Transparent lines are colour-coded by the individual.*


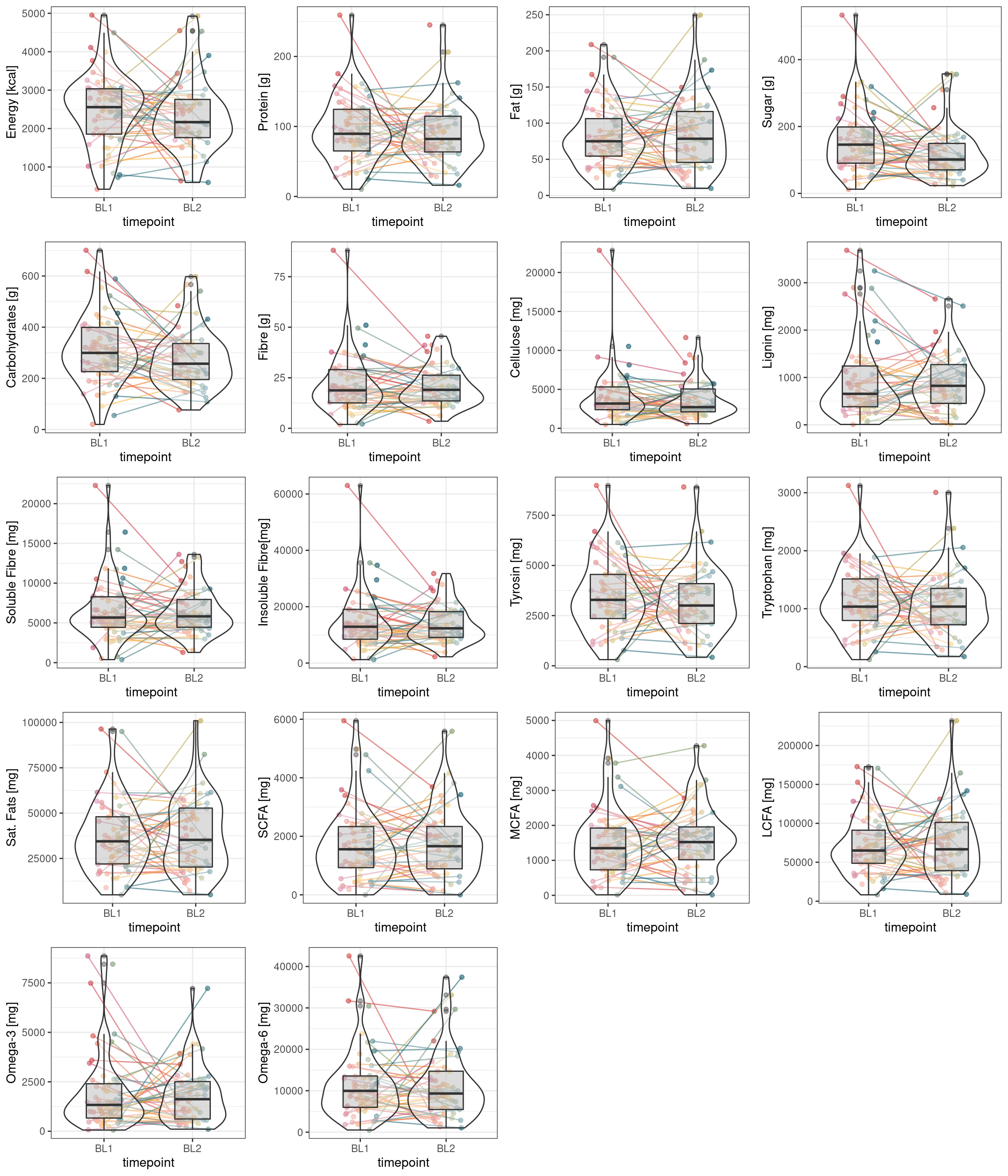


*SI-Figure 2: Computed nutrient values plotted across two timepoints for FFQ 24 hours. Individual lines are colour-coded by individual.*


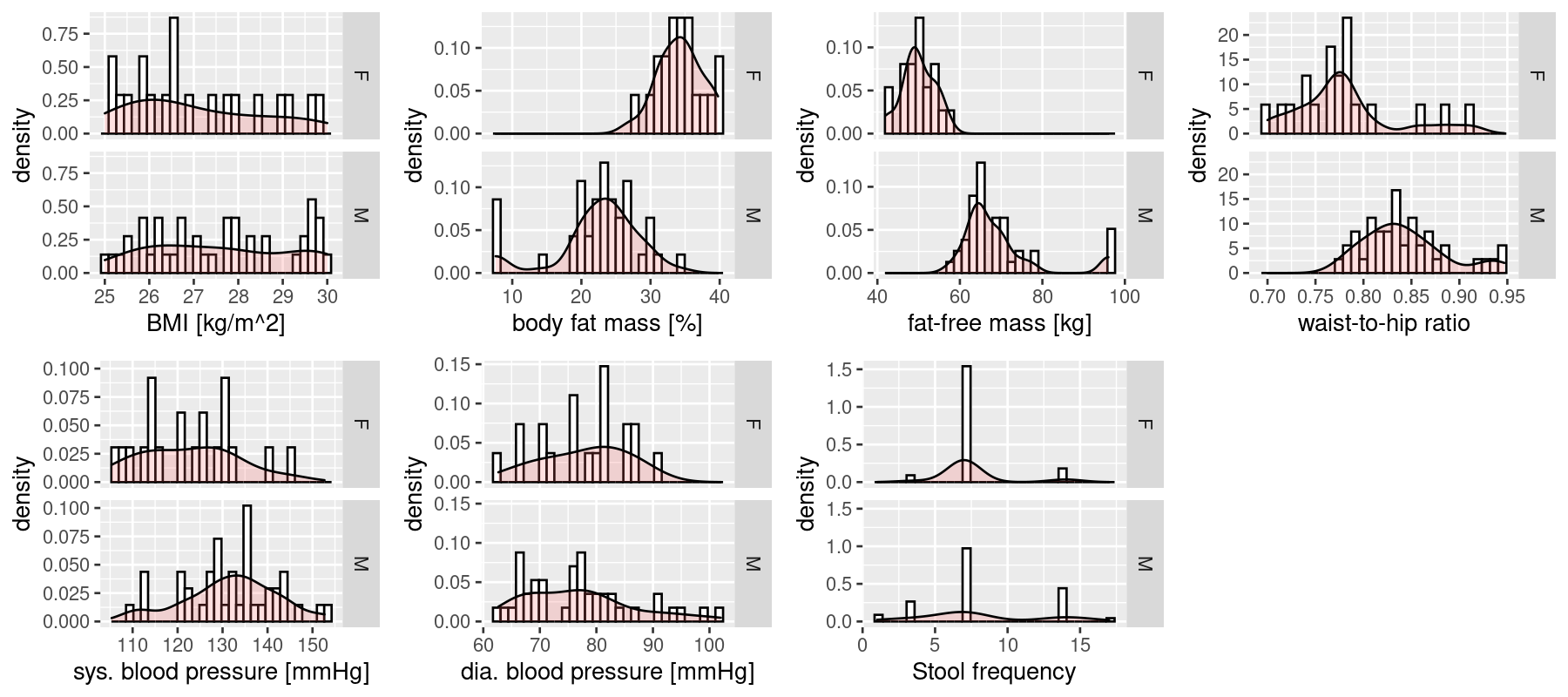


*SI-Figure 3: Distribution of anthropometric markers by sex. BL1 measures only for 59 individuals.*


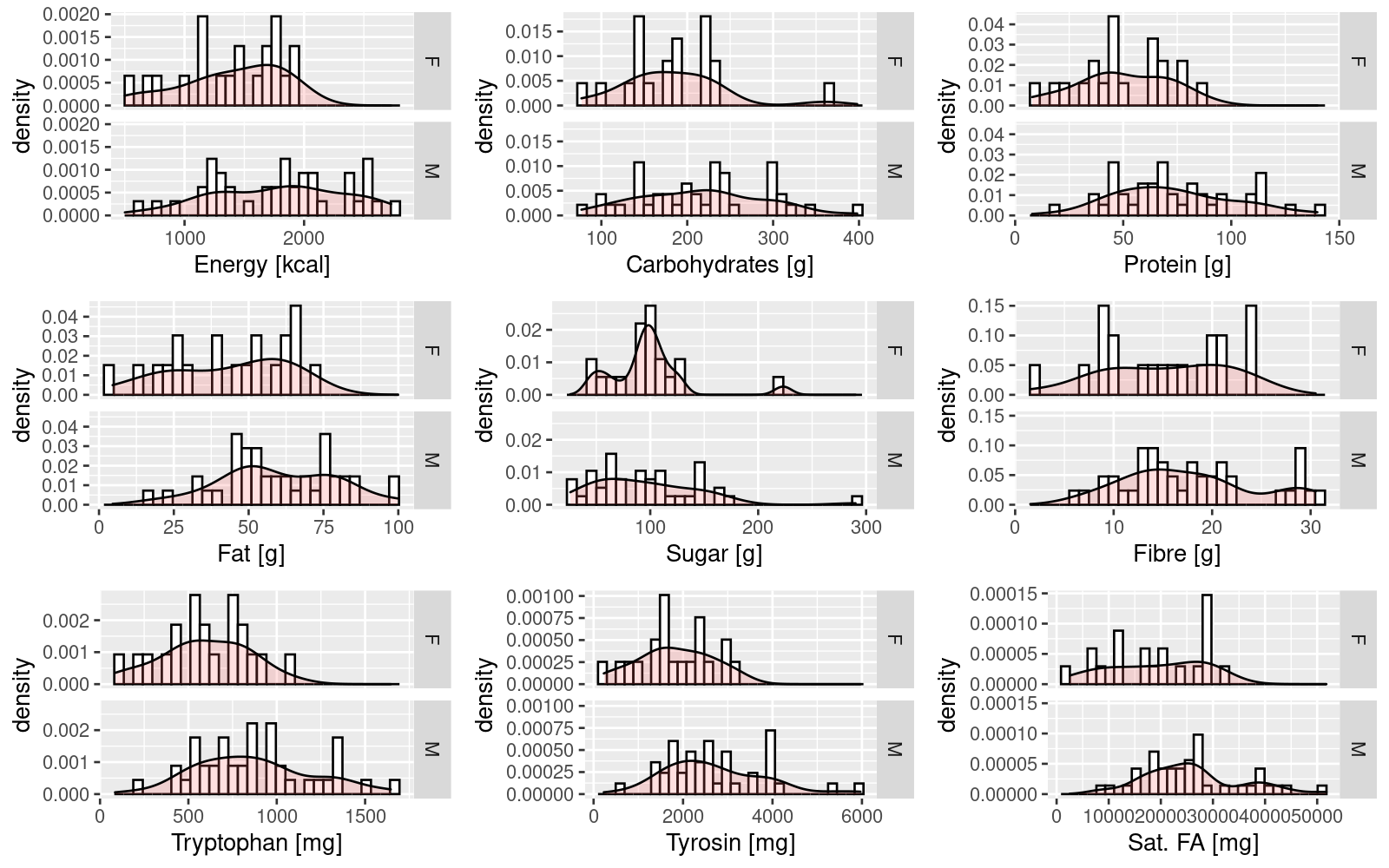


*SI-Figure 4: Distribution of nutrient values by sex. BL1 measures only for 59 individuals.*

*SI-Table 1: Computed, energy adjusted nutrient intake for a cross-sectional sample of adult women and men from two assessment days. P-values are indicated for standard 2-sample t.test.*

|  | **BL1 (N=114)** | **BL2 (N=97)** | **p value** |
| --- | --- | --- | --- |
| **Energy (kcal)** |  |  | 0.858 |
| Mean (SD) | 2032.044 (866.184) | 2012.728 (836.291) |  |
| Range | 421.137 - 4953.181 | 597.150 - 4925.400 |  |
| **Residuals of energy-adjusted Protein (g)** |  |  | 0.945 |
| Mean (SD) | -1.034 (24.040) | -1.243 (19.159) |  |
| Range | -91.499 - 112.953 | -55.871 - 80.426 |  |
| **Residuals of energy-adjusted Fat (g)** |  |  | 0.072 |
| Mean (SD) | -3.448 (21.261) | 1.500 (18.012) |  |
| Range | -94.738 - 36.037 | -44.042 - 66.545 |  |
| **Residuals of energy-adjusted Carbohydrates (g)** |  |  | 0.062 |
| Mean (SD) | 8.972 (53.269) | -4.240 (48.344) |  |
| Range | -181.612 - 233.276 | -130.042 - 157.033 |  |
| **Residuals of energy-adjusted Fiber (g)** |  |  | 0.683 |
| Mean (SD) | 0.266 (9.026) | -0.200 (7.223) |  |
| Range | -18.400 - 56.578 | -19.532 - 23.106 |  |
| **Residuals of energy-adjusted Sugar (g)** |  |  | 0.002 |
| Mean (SD) | 14.537 (55.442) | -8.475 (47.760) |  |
| Range | -126.434 - 299.330 | -207.066 - 129.546 |  |

### *SI-Table 2: Computed, energy adjusted nutrient intake for a cross-sectional sample of adult women and men for FFQ 24 hours and FFQ 7 days. P-values are indicated for standard 2-sample t.test.*

|  | 24h (N=103) | 7d (N=108) | p-value |
| --- | --- | --- | --- |
| **Energy (kcal)** |  |  | **< 0.001** |
| Mean (SD) | 2420.228 (957.410) | 1644.484 (499.709) |  |
| Range | 421.137 - 4953.181 | 500.869 - 2777.991 |  |
| **Residuals of energy-adjusted Protein (g)** |  |  | 0.616 |
| Mean (SD) | -1.906 (28.612) | -0.391 (12.575) |  |
| Range | -91.499 - 112.953 | -44.132 - 49.404 |  |
| **Residuals of energy-adjusted Fat (g)** |  |  | 0.696 |
| Mean (SD) | -1.725 (26.401) | -0.648 (10.743) |  |
| Range | -94.738 - 66.545 | -44.856 - 21.077 |  |
| **Residuals of energy-adjusted Carbohydrates (g)** |  |  | 0.678 |
| Mean (SD) | 4.410 (68.040) | 1.457 (27.599) |  |
| Range | -181.612 - 233.276 | -67.214 - 128.866 |  |
| **Residuals of energy-adjusted Fiber (g)** |  |  | 0.859 |
| Mean (SD) | -0.052 (10.720) | 0.151 (4.834) |  |
| Range | -19.532 - 56.578 | -13.242 - 17.709 |  |
| **Residuals of energy-adjusted Sugar (g)** |  |  | 0.556 |
| Mean (SD) | 6.174 (68.030) | 1.845 (33.630) |  |
| Range | -207.066 - 299.330 | -68.185 - 157.320 |  |

*SI-Table 3: Computed nutrient intake for a cross-sectional sample of adult men for FFQ 28 days. HFS = High Fat and Sugar Group, LFS = Low Fat and Sugar Group, OMN = omnivorous, VEG = vegetarian. Statistical comparison of the nutrients (except energy) was conducted with the residuals of the energy-adjusted values. P-values are indicated for standard 2-sample t.test.*

|  | OMN (N=59) | VEG (N=17) | p-value |
| --- | --- | --- | --- |
| **DFS group** |  |  |  |
| HFS | 32 (54.2%) | 3 (17.6%) |  |
| LFS | 27 (45.8%) | 14 (82.4%) |  |
| **HbA1c (mmol/mol)** |  |  | 0.096 |
| N-Miss | 1 | 0 |  |
| Mean (SD) | 32.977 (2.721) | 31.642 (3.353) |  |
| Range | 26.670 - 37.920 | 22.840 - 35.960 |  |
| **Energy (kcal)** |  |  | 0.062 |
| Mean (SD) | 2180.420 (802.195) | 1776.037 (673.133) |  |
| Range | 696.885 - 4053.625 | 664.193 - 3215.061 |  |
| **Protein (g)** |  |  | 0.415 |
| Mean (SD) | 87.379 (33.773) | 75.479 (46.826) |  |
| Range | 28.018 - 159.938 | 23.013 - 212.578 |  |
| **Fat (g)** |  |  | 0.372 |
| Mean (SD) | 78.926 (35.282) | 59.361 (26.734) |  |
| Range | 23.344 - 195.702 | 14.496 - 111.568 |  |
| **Carbohydrates (g)** |  |  | 0.526 |
| Mean (SD) | 259.794 (101.352) | 219.908 (93.607) |  |
| Range | 79.366 - 583.559 | 46.219 - 454.084 |  |
| **Fiber (g)** |  |  | **0.008** |
| Mean (SD) | 25.868 (11.889) | 29.036 (11.675) |  |
| Range | 8.686 - 62.150 | 11.956 - 53.830 |  |
| **Sugar (g)** |  |  | 0.620 |
| Mean (SD) | 111.784 (57.557) | 86.326 (35.620) |  |
| Range | 25.192 - 341.805 | 33.120 - 152.994 |  |

*SI-Table 4: Results of linear mixed models for normal-weight to obese sample (n=187, BMI: 18.6-36.4 kg/m2 M±SD: 25.9±2.8; GUT-BRAIN and GREADT). Prediction of BMI by residuals of energy-adjusted nutrient values. General equations:*

*H0: BMI ~ sex + age + MET total activity + (1/subject)*

*H1: BMI ~ nutrient value + sex + age + MET total activity + (1/subject)*

*Abbreviations: AIC: Akaike Information criterion; BIC: Bayesian information criterion*

| **(energy adjusted) nutrient value** | **Model** | **AIC** | **BIC** | **log likeli-hood** | **deviance** | **Chi-square** | **p(Chi-square)** | **b(nutrient value)** |
| --- | --- | --- | --- | --- | --- | --- | --- | --- |
|  | H0 | 727.96 | 747.22 | -357.98 | 715.9 |  |  |  |
| Energy [kcal] | H1 | 728.85 | 751.31 | -357.42 | 714.85 | 1.1143 | 0.2912 | -0.0001618 |
| Protein [g] | H1 | 729.77 | 752.23 | -357.88 | 715.77 | 0.1974 | 0.6568 | 0.002459 |
| Fat [g] | H1 | 728.76 | 751.22 | -357.38 | 714.76 | 1.2075 | 0.2718 | 0.006623 |
| Carbohydrates [g] | H1 | 727.83 | 750.29 | -356.91 | 713.83 | 2.1347 | 0.144 | -0.003632 |
| Fiber [g] | H1 | 727.58 | 750.05 | -356.79 | 713.58 | 2.3788 | 0.123 | -0.023514 |
| Sugar [g] | H1 | 729.93 | 752.40 | -357.97 | 715.93 | 0.0281 | 0.867 | 0.0003994 |

*SI-Table 5: Results of linear mixed models for normal-weight to obese sample (n=187, WHR: 0.65-0.98, M±SD: 0.81±0.05; GUT-BRAIN and GREADT). Prediction of WHR by residuals of energy-adjusted nutrient values. General equations:*

*H0: WHR ~ sex + age + MET total activity + (1/subject)*

*H1: WHR ~ nutrient value + sex + age + MET total activity + (1/subject)*

*Abbreviations: AIC: Akaike Information criterion; BIC: Bayesian information criterion*

| **(energy adjusted) nutrient value** | **Model** | **AIC** | **BIC** | **log likeli-hood** | **deviance** | **Chi-square** | **p(Chi-square)** | **b(nutrient value)** |
| --- | --- | --- | --- | --- | --- | --- | --- | --- |
|  | H0 | -615.70 | -596.45 | 313.85 | -627.70 |  |  |  |
| Energy [kcal] | H1 | -614.78 | -592.31 | 314.39 | -628.78 | 1.0741 | 0.3 | 0.0000060 |
| Protein [g] | H1 | -614.25 | -591.79 | 314.13 | -628.25 | 0.5479 | 0.4592 | -0.0001642 |
| Fat [g] | H1 | -614.35 | -591.88 | 314.18 | -628.35 | 0.6469 | 0.4212 | 0.0002189 |
| Carbohydrates [g] | H1 | -613.73 | -591.26 | 313.86 | -627.73 | 0.0224 | 0.8811 | -0.0000162 |
| Fiber [g] | H1 | -615.93 | -593.47 | 314.97 | -629.93 | 2.2281 | 0.1355 | -0.0007349 |
| Sugar [g] | H1 | -614.44 | -591.98 | 314.22 | -628.44 | 0.7374 | 0.3905 | 0.0000882 |

*SI-Table 6: Computed energy value for two exemplary food items and their respective food subgroups.*

| Food subgroup | FFQ-Answer | composition | Energy [kcal/100g] | Relative difference to reference value |
| --- | --- | --- | --- | --- |
| Chicken |  |  |  |  |
| only not fried chicken | 1 | NA | 197.17 | reference |
| ~ ¼ of chicken intake fried | 2 | 0.25*fried+ 0.75*not fried | 205.54 | +4% |
| ~ ½ of chicken intake fried | 3 | 0.5*fried+ 0.5*not fried | 213.92 | +8% |
| ~ ¾ of chicken intake fried | 4 | 0.75*fried+ 0.25*not fried | 222.29 | +13% |
| only fried chicken | 5 | NA | 230.67 | +17% |
|  |  |  |  | up to +17% |
| Coffee |  |  |  |  |
| without sugar | 1 | NA | 2 | reference |
| 1 teaspoon of sugar | NA | 1 teaspoon | 20.25 | NA |
| with 1 teaspoon of sugar | 2 | coffee + 1 teaspoon | 22.25 | +1112.5% |
| with 2 teaspoons of sugar | 3 | coffee + 2 teaspoons | 42.25 | +2112.5% |
| with 3 teaspoons of sugar | 4 | coffee + 3 teaspoons | 62.25 | +3112.5% |
|  |  |  |  | up to +3112.5% |

*NB: Food items “Chicken” and its different preparation types and “Coffee” with different amounts of sugar. Last column emphasizes the difference in energy value if the additional information obtained by food subgroup questions was disregarded in the nutrient calculation as it is disregarded in the original scoring.*
